# Culturally and structurally embedded pathways to youth self-harm in Rwanda: perspectives from young people, parents, and healthcare providers

**DOI:** 10.1186/s40359-025-03676-y

**Published:** 2025-11-22

**Authors:** Faith Martin, Joseph Kalisa, Belise Blandine Isingizwe, Yves Gashugi, Faith Cheonga, Evangeline Ishimwe, Prince Uwizeye, Sarah Wicker, Shu Yi Ong, Darius Gishoma, Vincent Sezibera

**Affiliations:** 1 Department of Psychology, University of Bath, Claverton Down, Bath, BA2 7AY UK; 2 Centre for Mental Health, College of Medicine and Health Sciences University of Rwanda, Po Box. 3286, Kigali, Rwanda; 3 Rwanda Psychological Society, KK493 St, Kigali, Rwanda; 4 Department of Public Health, Aarhus University, Aarhus, Denmark; 5 School of Psychology, Cardiff University, Tower Building 70 Park Place, Cardiff, CF10 3AT UK; 6 Rwanda Biomedical Centre, KG 644 St, P.O. Box 7162, Kigali, Rwanda

**Keywords:** Youth, Self-harm, Culture, Qualitative, Structural determinants

## Abstract

**Background:**

Self-harm (with and without fatal intent) among young people in low- and middle-income countries (LMICs) remain under-researched despite disproportionately high rates. Little is known about the social, cultural, and psychological factors influencing these behaviours in settings such as Rwanda. The study aimed to explore how young people in Rwanda, their parents, and healthcare providers perceive and experience self-harm, including the psychological, social, and cultural factors that contribute to self-harm.

**Methods:**

This qualitative study used a phenomenologically informed approach. Semi-structured interviews were held with 102 participants across two districts of Rwanda: Gasabo (urban) and Nyagatare (rural), which have high prevalence of self-harm/suicidality, including young people with and without self-harm experience, parents of young people with and without such experience, and healthcare professionals. Data were analysed thematically, using iterative coding, with triangulation and member-checking to enhance rigour.

**Results:**

Five themes emerged: 1) Diverse triggers and reasons—including family conflict, abuse, poverty, peer dynamics, and school-related stress; 2) Build-up of emotional and psychological distress—highlighting feelings of entrapment, isolation, and worthlessness; 3) Functions and characteristics of self-harm, ranging from emotional regulation to communication of distress; 4) Maintenance and cessation, showing the role of coping strategies, social support, and barriers to seeking help; and 5) Duality of community responses, where community responses both exacerbated stigma and provided support. Self-harm was shaped by cultural beliefs, stigma, family and social structures, and poverty, challenging individualistic framings of self-harm. Reflexive insights highlight the importance of team communication during cross-cultural research and provide practical strategies.

**Conclusions:**

Findings extend existing psychological theories by evidencing culturally embedded pathways to youth self-harm, where distress is produced and sustained through a mixture of individual experience, structural hardship, social exclusion, and cultural belief systems. Effective prevention requires cross-sectoral strategies addressing poverty, education, family support, and community mental health. Community-based approaches fostering emotional expression and reducing stigma could play a crucial role in prevention and recovery. The insights generated are transferable to other LMICs facing similar social and structural challenges.

**Supplementary Information:**

The online version contains supplementary material available at 10.1186/s40359-025-03676-y.

## Introduction

Suicide is the third leading cause of death globally for 15–19 year olds and 88% of adolescents who die by suicide globally are from low-middle income countries (LMICs) [1]. Suicide prevention is a sustainable development goal [2]. Epidemiological data on self-harm, particularly non-fatal self-harm, is limited in some regions, however appears common in LMICs [3].

The terminology demands clarification. “Self-harm” refers to intentional acts to harm oneself through self-poisoning or injury, regardless of intended or actual fatality [4]. Where self-harm has a fatal outcome, this is suicide [5]. In some contexts, there is an attempt to subdivide, based on intention to die or not [4, 5]. In both high-income countries (HIC) and LMICs, the distinction between self-harm and suicide is contested, as intentionality and motivations may not be dichotomous [5–8]. The function of “non-suicidal self-injury” to regulate emotions is established in high-income countries (HIC) [9–11], but less clear in other settings [6, 12]. Importantly, in many LMICs, including Rwanda, cultural understandings of distress, emotion, and behaviour may not map neatly onto Western psychiatric categories [6]. Here, we use “self-harm” as an inclusive term encompassing all forms of intentional self-injury. Where intent is clearly specified, either by participants or in cited literature, we report it explicitly.

Despite the high risk to adolescents living in LMICs, research is dominated by work from HIC [13]. This is concerning, given reports of rising rates, and the presence in LMIC of the intersection of poverty, trauma, and limited mental health infrastructure in some settings [14], together with high levels of stigma around mental health and self-harm[15, 16]. A recent systematic review of research into self-harm in young people in sub-Saharan Africa (SSA) reported they found “no substantial study where participants [young people] were asked in detail about reasons for self-harm” [18, p14], highlighting the need for research sensitive to young people’s lives and family structures in SSA. For example, extended family networks and the close involvement of community members have meant a need to tailor interventions for young people, showing the importance of family context to how we understand and support wellbeing [17–19]. Indeed, an important qualitative study by Quarshie et al. highlighted how self-harm in adolescents is deeply situated within family, social, and cultural context [20]. Although current research in SSA has begun exploring young people’s perceptions [21–24], little research has explored protective factors, the role of social norms, or intergenerational perspectives, particularly including how parents and community members perceive youth self-harm. This research is important to inform intervention development.

In Rwanda, around 60% of the 13 million population are aged under 24 years [25]. A population based, cross-sectional study of youth in 2019 found that 4.7% females and 1.4% males aged 20–35 had “attempted suicide” in the preceding month [26]. In the previous 6 months, 20.6% of 10–17-year-olds sampled from schools in 2015 had experienced suicidal ideation; 18.3% did at least one suicidal behaviour (irrespective of intention to die) [27]. Rates appear higher in urban compared to rural regions, based on a 2021 survey [28]. Adolescents in Rwanda face a complex constellation of risk factors for self-harm, including intergenerational trauma stemming from the 1994 Genocide against the Tutsi, socioeconomic hardship, living with HIV, and severe physical punishment [27]. Parental mental health plays a crucial role: research shows that parents’ own traumatic experiences affect adolescents' well-being through disrupted emotion regulation and parenting styles, linked to inter-generational trauma [28–32]. Within these dynamics, family relationships and expectations emerge as both protective and risk factors, especially in contexts where traditional values and familial obligation are emphasized [33]. Parents perceptions are vital, as their child’s distress may impact them [32, 34], and parents’ responses may shape their young person’s wellbeing and access to care [35, 36]. While Rwanda’s context is specific, findings may have relevance for other post-conflict, low-income settings, featuring collectivist norms and spiritual interpretations of mental distress [36, 37].

This study aimed to explore perceptions, experiences and meaning-making of self-harm in young people in Rwanda, including view-points from young people both with and without direct experience, as well as by their parents and healthcare providers. Using in-depth qualitative interviews, the study examined the social, cultural and psychological factors that increase or reduce the risk of self-harm, and how these factors are perceived across different participant groups. Comparing data from different participant groups allowed exploration of their commonalities and differences. This study is the first to explore youth self-harm in Rwanda, examining multiple perspectives, and among the first in sub-Saharan Africa.

## Methods

Phenomenologically informed inductive thematic analysis was employed [38], in order to meet our aims to understand the subjective experiences of youth and parents as they experience and make sense of self-harm [39]. This study reports the findings arising from our initial open coding. We were guided by guidelines for reporting qualitative research (Additional File 1 for checklist) [40].

### Participants and setting

Participants received full written information about the study and research team, including the right to withdraw, confidentiality, and information about support. For those unable to read, the information was read to them, with frequent summaries and opportunities for questions. This allowed informed decision-making irrespective of literacy level. Informed consent was provided by all participants. Written consent was recorded, with support to document consent for those unable to write. The research received approval from the ethical institutional review boards of the College of Medicine and Health Sciences, University of Rwanda (368/CMHS_IRB/2023) and the School of Psychology, Cardiff University (EC.23.09.12.6843.).

We recruited five different groups of participants:

1. YP + : Young people (16–22) with self-harm experience, engaged in mental health support.
2. YP-: Young people (16–22) with stated they had no self-harm experience.
3. P + : Parents of young people (16–22) who had self-harmed, including parents of those who had died by suicide at least six months prior.
4. P-: Parents of young people (16–22) with no known self-harm experience.
5. HCP: Health-care professionals and others working with people in relation to their health, (e.g., clinical psychologists, psychiatric nurses, community health workers, hereafter collectively referred to as HCPs for ease) with experience supporting families affected by self-harm. This group were included for their direct experiences of supporting people, but also to explore their view-points and experiences which may have related to being in the health-care system and witnessing the experiences of multiple young people/families.

Participants were recruited across one urban (Gasabo in the capital Kigali, in central Rwanda) and one rural (Nyagatare, towards the north-east) region to capture diversity. Data from the Ministry of Health’s Rwanda Biomedical Centre (RBC) in 2019 ranked these regions as having the highest prevalence of suicide and mental health difficulties, increasing from previous survey years. Participants were recruited via health-care facilities: Bugaragara Health Center, Gatunda District Hospital and Cyabayaga Health Center in Nyagatare; Kinyinya Health Centre, Gikomero health center, Kibagabaga hospital, and via the mental health peer support organisation “OPROMAMER” in Gasabo. These facilities provide services across the two selected districts, with catchment areas covering where people live. Smaller health centres (as opposed to the major District Hospitals) were included as these are typically closer to potential participants’ homes. Sampling was purposive, to cover our five participant groups, with the intention to recruit equal urban/rural and participants identifying as male and female.

Recruitment was supported by Rwanda Biomedical Centre (“RBC”, a national institution of the government of Rwanda, associated with the Ministry of Health), who gave us permission to contact healthcare facilities and provided a “letter” (sometimes emailed) asking the facilities to support recruitment. HCPs searched records of individuals who had themselves (YP +) or had children who had previously engaged in self-harm or suicidal behaviours (P +) and informed them about our study. With participant consent, contact details were shared with the research team, who followed-up directly, or liaised via the mental health professionals. Records were not kept by the health professionals re: people who declined to be contacted by the research team. Of those who agreed to be contacted by the research team, all consented to take part, however two subsequently declined following advice from family members, which may signal the sensitivity of the topic. The research team had no previous relationships with the participants, and participants were aware they were being approached by people in a research role specifically to conduct interviews only. Participants who were parents or young people with no direct experience of self-harm in young people (P- and YP-) were recruited through local leaders (“village leaders”, elected by the community, who live in the community, know the local people, and would be aware of cases of suicide or serious self-harm requiring medical intervention owing to the way communities share information). The researchers conducted a screening conversation, running through the eligibility criteria, to check eligibility.

### Data collection

Data were collected through one-on-one, semi-structured interview conducted in Kinyarwanda, typically lasting 45–60 min. Initially, we planned two interviews with young participants to build rapport and gather life histories. However, piloting and community feedback showed a single interview was sufficient, as participants felt comfortable sharing due to the interview setting and the mental health professional status of interviewers. Topic guides (examples in Additional File 2) covered: a) conceptualisation of self-harm; b) experiences of self-harm; c) perceived causes, drivers, risk, and protective factors; d) support access and unmet needs; and e) community and parental responses. The topic guides were developed with the research team initially in English, then translated to Kinyarwanda during a team workshop in Rwanda, with discussion of terms to use to capture the relevance concepts in Kinyarwanda. This topic guide was piloted with colleagues and subsequently with three eligible participants, resulting in minor changes to improve clarity of phrasing.

The interviews were conducted at the healthcare and “OPROMAMER” (a mental health peer support organisation) offices, to ensure participants were in a private space, to maintain confidentiality. Input from public representatives during study design informed this, as they reported was conducive to being able to openly share their viewpoints. Data were collected by one male and one female researcher, (YG and BBI), both Rwandan Clinical Psychologists with research experience and training in conducting qualitative research, and role-plays of the interviews to enhance skills. The researchers collecting data were Clinical Psychologists, as were the majority of the wider team.

For participants who disclosed a current risk of self-harm, or other risks (e.g. domestic violence), their information was recorded using a specially designed form, covering contact information, personal details, reported indications of risk. Information was shared (see Ethics section).

To ensure researcher well-being, we implemented regular supervision sessions with an experienced clinical supervisor not directly involved with the research for emotional support. These sessions helped those collecting the data to identify and manage potential issues early, fostering a supportive and resilient research environment.

### Data analysis

Data were verbatim transcribed initially into Kinyarwanda, and de-identified at this point (removal of any details of names, places, or specific details that would identify either the participant or a third party). To ensure translation accuracy and conceptual equivalence, selective back-translation was used [41, 42]. Five transcripts were independently back-translated by a bilingual researcher (BBI, YG, EI, JK, PU). Each was reviewed by another bi-lingual researcher (JK or VS). A translation validation meeting was held (BBI, YG, EI, JK, PU, VS) to review side-by-side the Kinyarwanda and English versions, with particular discussion around difficult and/or key-words. A “word-bank” of hard to translate phrases was compiled and reviewed by the University of Rwanda’s language department to find appropriate equivalence in English. This was added to during the translation process, with back-translation occurring for other transcripts where difficulties were encountered. The insights from the validation meeting were used to guide all other translation. This approach balances methodological rigour with feasibility in large-scale qualitative studies [42]. Rather than aiming for direct linguistic equivalence, we focused on meaning and contextual nuance [41, 42].

Coding was conducted without the use of any a priori framework: inductive thematic analysis was used [38], focused on the experiences described in the data, important as researchers came from different cultural backgrounds. Researchers began with repeated readings of the transcripts to achieve familiarisation, then coded line-by-line to capture meaning units relevant to experiences of self-harm. Coding was done “by hand” in Microsoft Word and paper notes by the majority of the team, with NVivo [43] and MAXQDA software [44] used by FM and SW respectively to support organisation of codes to themes. Codes were compared within and across transcripts, and iteratively grouped into broader code families, by individual researchers and then during team discussion. From these code families, themes were derived through an interpretative process that was intended to move beyond description to identify patterned meaning across the interviews in the dataset. This involved analysis of both semantic content and latent meanings. Candidate themes were considered against the data, focusing on coherence and distinctiveness in themes. Next, analysis of the same group of participants was discussed in pairs, with an emphasis of reflexive observations noted by researchers during their analysis process. Analysis was conducted by eight team members from Rwanda, Malawi, United Kingdom, Malaysia, and France. Finally, the team met in person for a week, to work together on the coding, sharing and discussing codes and themes from their own analysis and those created by colleagues working on data from other participant groups, refining the themes. This provided investigator triangulation to help identify and address biases.

The refined themes were then presented to a group of experts, including participants who volunteered to attend, health-care professionals, religious leaders, and policy makers, to gain their feedback as a form of both member checking and triangulation. The experts felt findings relating to family dynamics/conflict were particularly significant, leading to further analysis. Participant validation or “member checking” was also performed, to enhance credibility [45]. This was tailored to be accessible to our participants. We presented key themes in the format of a pre-recorded, scripted drama of a fictional (based on our themes) adolescent girl and her family. This prompted discussion in groups of YP + and YP-, and P + and P-. Field notes, from these meetings in the two districts, were discussed as a team, validating the analysis without necessitating further changes.

## Results

Interviews were conducted with 102 participants in total, as detailed in Table 1.

**Table 1:** Numbers of participants in each group by region and gender

**Participant group**	**Urban region: Gasabo**		**Rural region: Nyagatare**		**Total**
	**Male**	**Female**	**Male**	**Female**	
Health care professionals (or others working to support health e.g. community health workers) (HCPs)	0	3	2	1	6
Young person with experience of suicide/self-harm (YP +)	3	9	5	7	24
Young person with no experience declared at recruitment (YP-)	4	8	1	11	24
Parents of young person with known experience (P +)	1	11	5	7	24
Parents of young person with no known experience of suicide/self-harm (P-)	6	5	4	9	24
Total	14	36	17	35	102

### Themes

Here, we focused on the key features of the experiences and perceptions of self-harm in young people in Rwanda, we identified five themes: 1) Diverse triggers and reasons, setting out the perceived causes and purpose of self-harm, which are presented under a range of topics; 2) build up of emotional and psychological distress, highlighting the experience of distress that led to self-harm; 3) the functions and characteristics of self-harm, examining ideas about what these behaviours “do” for the young person; 4) maintenance and cessation, analysing the factors linked to whether these behaviours continue or stop; and 5) the duality of community perceptions and responses, evidencing how the community can be both harmful and helpful for the young person. The coding tree is provided in Table 2. In many cases, there was agreement or similar ranges of ideas between the different participant groups (YP +, YP-, P +, P-, and HCPs). For analyses where this was not the case, we have clearly set named this. In other cases, the reader should assume there were no divergences in theme expression clearly linked to participant grouping.

**Table 2:** Coding tree: thematic analysis of youth self-harm in Rwanda

Theme and subthemes (where present)	Main codes
**Theme 1: Diverse Triggers and Reasons**	
Abuse and Neglect	Types of experience
Family structures and conflict, including unplanned pregnancy	Conflict
	Changes in family structure
	Parental refection and lack of love
	Lack of emotional support
	Unplanned Pregnancy
Poverty and Deprivation	Poverty links to other stressors
	Cumulative emotional impact
School and Academic Pressure	Impact of challenges in access to education
	Gendered access to education and opportunity
Peer and Romantic Relationships	Emotional impact of relationship challenges
	Presence and lack of supportive relationships
	Peer pressure
**Theme 2: Emotional and Psychological Distress**	
	Overwhelm
	Shame and bitterness
	Meaninglessness
	Worthlessness
	Hopelessness
	Isolation
**Theme 3: Functions of Self-Harm**	
	Methods
	End unbearable distress
	Avoid social consequences
	Managing overwhelm/emotion regulation
	Interpersonal and relational functions
**Theme 4: Maintenance and Cessation**	
Barriers to cessation	Awareness of services and support
Pathways to cessation	Lack social support
	Coping strategies
	Community support
	Formal support
**Theme 5: Duality of Community Responses**	
Stigma, silence, harmful judgment	Stigma as causal trigger
Containment, care, and connection	Community and cultural beliefs about self-harm
	Unacceptable nature of self-harm
	Reflection on wider family
	Range from advice to punishment
	Positive support from range of community members
	Practical advice
	Peer support
	Changes and opportunities for family support

**Fig. 1:**
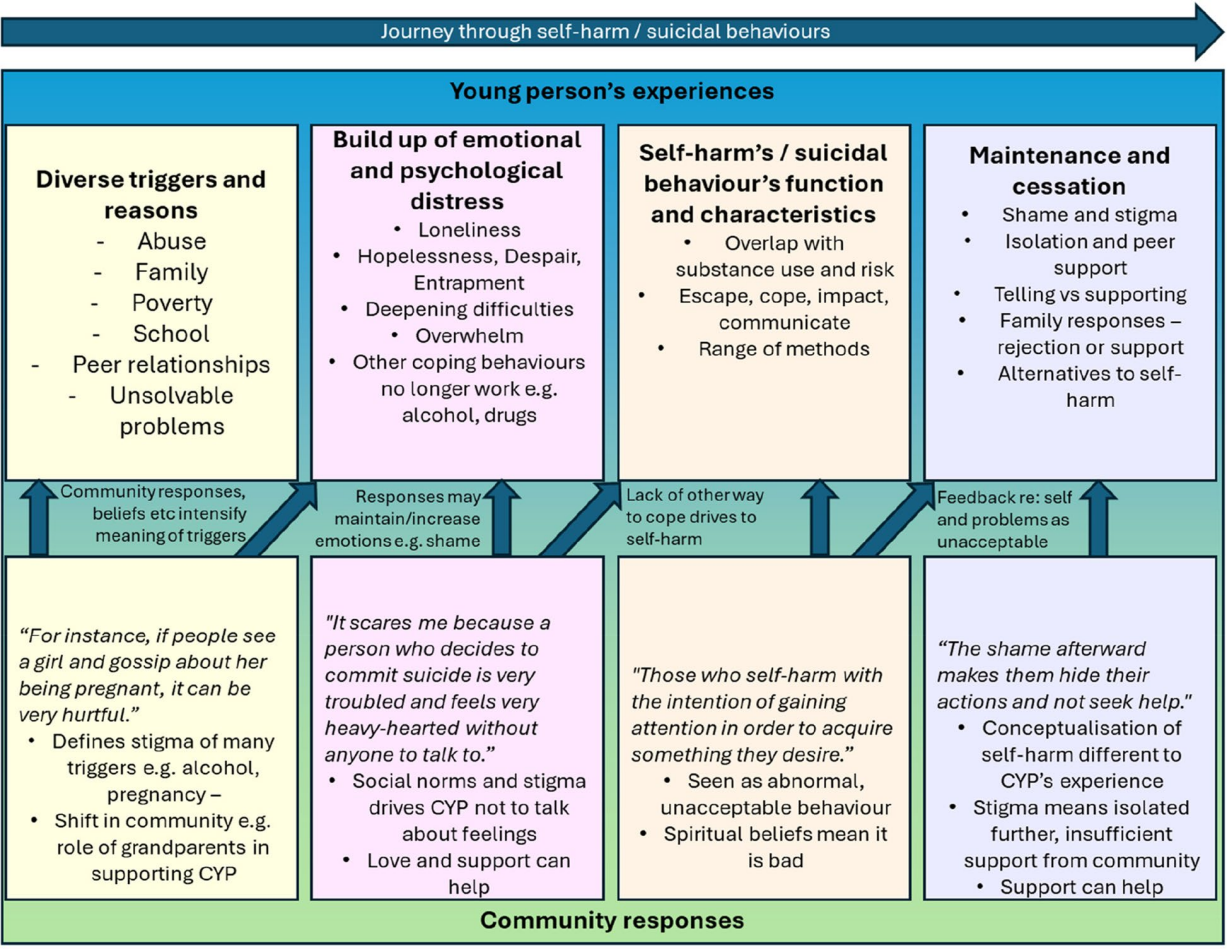
Illustration of the link between the themes and community responses

The themes, as we derived them, represent something of the journey from cause and intrapersonal experience, to enacting the behaviour and continuing or stopping this behaviour. No a priori framework relating to a journey influenced the analysis, however the structure of the topic guide may have influenced how participants described their experiences. Community responses influenced the experience of all other elements. Figure 1 seeks to illustrate this, with a summary of the first four themes, and details of how community responses impact each theme.

For each theme and sub-theme, illustrative quotes are provided in Table 3, in addition to further in-text quotes.

**Table 3:** Showing participants quotes for Theme 1 and additional illustrative quotes for Themes 2–5

Theme	Subtheme/topic	Quote (participant identifier)
**Diverse Triggers and Reasons**	Abuse	“painful memories or thoughts, which mentally disturb me” (YP + YN0060)
	Family structures and conflict, including unplanned pregnancy	“Do you know how it feels when you’re sleeping in another person’s house, and they come and tell you to get out?" (P + BG 0162)
		"It would be better if I committed suicide and ended it all because I feel like I'm living as someone who doesn't exist, especially in front of my parent, who I feel rejects me in favour of other children." (YP + YN 0057)
		“What saddened me in my childhood is that we have a family, but we were not loved in the family” (YP + YN 0052)
		“My father has been addicted to substance abuse and he was supposed to be my advisor” (YP + YG 0082)
		“The child might be mistreated internally, but externally it appears as though they are well taken care of, with people saying, "That child is well taken care of by their stepmother," while inside the child is suffering. You might hear that [girl] committed suicide and wonder why, thinking that her stepmother treated her well.” (YP-BG0134)
		“We [family] never sat together and talked about things. We only talked about what we will do the next day. We never have deep conversation" (YP + YN001) “Our mother never showed tiredness in raising four children. She never gave up or neglected us in the midst of her fights with our dad” (YP + BG 0132)
		“At family gatherings at home, once everyone has eaten, adults often engage in conversations with their children. This can be a crucial time when a child feels comfortable enough to discuss their problems, potentially preventing them from harming themselves, or considering suicide.” (HCP G)
		“I think it’s caused by problems someone is facing, like an unplanned pregnancy…or family problems, or if life feels too difficult and you see no way out… They might feel like their parents will be furious or even kill them, so they choose to end their own life instead.” (YP-BG0142)
	Poverty	“She was hurt by how she could not eat during the day and at night, had no clothes when she should have received all that, and she was also hurt by how we had to rent a house. That was a problem because I cannot afford to get the money to pay for a house.” (P + BG 0162)
		“Born into poverty, they can’t get what they want. That’s when they leave home to work as housemaids or in bars, and working in a bar requires drinking alcohol and smoking. So, when they are in those activities, they often don’t know what they are doing… then they go to their families, they are rejected, when they go to the person who impregnated them, they are denied, when they go to the community, they are rejected, that’s when they think, if I take something strong, I’ll die, and it will be over.” (YP-BN0063)
	School related challenges	“Self-harming can happen when a person feels confined and wants to go to school” (YP-BN0056)
		“Not having all the necessary requirements, which causes shame. For example, if a student needs seven or eight notebooks but can only afford one, this may trigger the child to feel unwanted in the world. This may lead to suicide or self-harm.” (HCP B)
		“I was not able to memorize anything…Later….I first attempted suicide by dropping myself into a river” (YP + YG 082)
		“Growing up with many siblings, sometimes parents still hold old-mindset views and might say girls shouldn’t go to school while boys should. A person might think of suicide, asking why they can’t go to school while their brothers can.” (YP-BN0052)
	Peer relationships	“Then he started talking about my sister who committed suicide, as someone who knew some of my secrets. Then I went to a store and bought some liquor to take home. That moment hurt me a lot.” (YP + BN 0074) “Maybe that’s what led to suicide attempts because if I had someone to share with, I wouldn’t do it.” (YP + YN 0052)
		"We were lovers, and later we separated. In separation, that’s when I had suicidal ideas” YP + YN 0052
		“He or she can't think of committing suicide on their own; it depends on the society they grow up in. They may seek advice from friends on what to do, and if their friends tell them to commit suicide, they may follow that advice.” (YP-YG0073)
**Theme 2: Build-up of emotional and psychological distress**	Overwhelm	“When a person takes a decision to commit suicide [meaning to die by suicide], I feel that life has overwhelmed them, that life has been so hard for them.” YP + BG 0132”
	Meaninglessness	[Describing feelings when she was self-harming " I feel like there's nothing worth living for. I feel like I have nothing good in my life…." YP + YN 0057
	Worthlessness	“I kept questioning why I was here in this life, because I couldn’t support my mother or my siblings with anything as the first-born, which made me self-hate” YP + BG 081
**Theme 3: Functions and characteristics of self-harm**	Managing overwhelm	“The triggering factor for self-harm [specifically asked re: without intention to die] is feeling overwhelmed and self-hatred, often stemming from unfulfilled desires. No one would decide to commit self-harm out of comfort… Self-harm typically arises from losing something or feeling directionless. When you gain something and then lose it, you may hate yourself and feel worthless.” (P-YN043)
**Theme 4: Maintenance and cessation of self-harm**	Barriers to cessation	“The challenge was how I was going to share my hardships with someone I didn’t know….and I had been carrying it since my childhood” (YP + YG 0078) Some felt simply that no-one could help in any case: "I felt that everyone was like my parent, and no one else could help me no matter what, I felt it wouldn't work…" YP + YN 0057
	Pathways to cessation: coping strategies	"I would sometimes be afraid to go home to avoid being beaten… Before going home from school, I would play football to try and find some happiness." (YP + YN 0057) “I thought, if I die without having a child…I would say, let me live for that child and work hard to provide for them… Those thoughts left me” (YP + BN 0074)
	Pathways to cessation: community support	“When there are things that have hurt me and end my capabilities, I find someone I can confide in, and they may give me advice, and I may feel unburdened” (YP + BG 0132)
	Pathways to cessation: formal support	“I went there [hospital] without sleeping, even though they gave me medications, but the dialogue helped me feel better” (YP + YG 0078) “Because I've received counseling many times, I've been able to move forward” YP + BG 0112
**Theme 5: Duality of community perceptions and responses—harmful and helpful**	Stigma, silence and harmful judgement	“[talking about deciding if to talk to a friend who had tried to kill themselves] …you can approach and counsel her that suicide is not a solution and is not acceptable” (YP-YG0071) “The reason they [parents] might feel ashamed is because the child thinks about committing suicide due to something based by the parent. When the neighbours see the child attempted suicide because of the parents’ mistreatment, they will say it was the parent who caused it.” (YP- BN0063) “They [community] give them [the parents] advice and tell them that the one who commits suicide does not go to heaven” (YP + BN 0099) “Most of them [fellow church members] feel that you [the parent] brought it upon yourself and people leave you there as if you’ve brought the horror upon yourself” (P + BN0086)
	Containment, care and connection	“They [school teachers] gave me advice and they sent me to bring what I was going to use to commit suicide and I gave it to them” YP + BN 0099 “They counselled me and conveyed me to the hospital.” YP + YG 0082

### Theme 1: Diverse triggers and reasons

Multiple drivers and triggers of self-harm were described by participants. Whilst mention was made of mental illness and drug abuse as primary causes of self-harm, typically this was within the context of complex, inter-related factors, as more primary reasons or triggers. These were predominately different types of “hardship” and were often interlinked. The commonly described types or elements that were seen as triggers or reasons were abuse; family issues; poverty; school challenges; and peer relationships.

#### Abuse

Abuse was a significant trigger for self-harm and suicide, described by all participant groups, though primarily by those with direct experience (YP + and P +). To maintain respect and anonymity, detailed quotes have been limited. Participants described physical, sexual, and psychological abuse, along with neglect. Examples included rape by family members, often disbelieved or ignored (YP + YG0078); beatings with household objects (YP + YN0057); chemical burns (P + YG85); and being locked up (P + YG85). Some participants used terms like “mistreatment” or “abused” without elaborating. Significant, sustained, and serious abuse was present in many YP + accounts, suggesting a substantial role of trauma and associated psychological challenges from many young people. Some participants described childhood traumatic stress reactions.

#### Family structures and conflict, including unplanned pregnancy

Family dynamics and conflict were mentioned by all participant groups. YP + named the impact of abusive family relationships, parental rejection, and lack of psychological, emotional and/or practical support from parents. These led to the build-up of emotional and psychological distress, explored further in the next theme. One YP + explained how their friend, struggling with family rejection and an abusive stepmother led her to attempt to die by suicide (*YP* + *BG 0112*).

Changes in family structure, such as remarriage, parental abandonment, or parental death, were described as causing traumatic and unmanageable situations, at times leading to familial conflict, psychological abuse and potential neglect. Parental remarriage led to shifts in living circumstances that could create distress. Parental rejection, experiences of not being loved, and parents being unable to provide love (e.g., owing to the parents’ mental health) were all also named*.* Some young people mentioned that their parents struggled with mental health issues such as drug and alcohol addictions which made them either abusive or neglect the parent. Young people described how the impact of family conflict was hidden from others, perhaps explaining why this was less commonly and strongly discussed by parents and health-care professionals.

A lack of emotional support or a space for expression, particularly within the family, was a contributory factor to self-harm. Conversely, love and protection from one parent, typically the mother, could be protective. A HCP explained that this type of support could happen during everyday contact at home.

Unplanned pregnancy in young people especially teenagers was another significant issue raised by participants across all five groups of participants. While some parents offered support, others viewed it as socially undesirable. For young people, unplanned pregnancy often felt like an insurmountable problem, leading to overwhelming stress and suicidal ideation.

#### Poverty

Family conflict and unplanned pregnancies were both linked to poverty. Changes in family structure, living arrangements, and overall family stress were associated to lack of material resources. Sex work, also linked to unplanned pregnancy, (P-YG67, P-YN46), was linked to poverty. Participants from each group described how socio-economic hardships, including poverty and malnourishment, could drive self-harm, often via overwhelm (e.g. YP + YN 0034). Parents of young people without self-harm (P-) expressed the role of orphanhood and poverty, which were often linked, as primary causes of self-harm. This connection was less emphasised in other participant groups. Many P + and YP + gave examples of situations of poverty creating significant difficulties in life.

Participants (particularly YP + and YP-) spoke about how poverty created a cumulative impact, leading to feelings of hopelessness and the perception that life’s challenges were impossible to overcome, which in some cases led to self-harm with fatal intent.

#### School related challenges

Access to education was crucial to both YP + and YP-, with many discussing lack of access to education, as parents/caregivers could not or would not fund this. Lack of access was common in YP + within single parent households. Challenges in accessing education was linked directly to self-harm. However, even if school was accessed, being unable to meet additional costs for equipment was associated to self-harm. Furthermore, given the highly prized nature of education, perceived failure at school was another trigger.

Gender differences were apparent in relation to access to school, increasing distress amongst girls. The lack of opportunity constrained the young girl’s sense of future, potentially linking to feeling trapped, as explored in the next theme concerning build-up of emotional and psychological distress.

#### Peer relationships

Features of peer relationships were linked to self-harm. YP + but not parents, described the emotional distress associated to breakdown of romantic relationships. The role of peer influence was described by YP + and YP-. They spoke about getting involved with drugs, alcohol, and delinquent behaviours as they narrated the journey towards self-harm. Some young people saw a lack of supportive peer relationships as increasing risk of self-harm. Parents also talked about peer pressures and influences, however unlike the young people, they did not reflect on challenges in relationships themselves.

### Theme 2: Build-up of emotional and psychological distress

Participants described how distressing emotions and psychological responses built up in reaction to the triggers outlined in the previous theme. Often, life challenges compounded each other, amplifying distress. For example, several young people explained how the death of their mother led not only to grief but also to practical challenges, such as changes in home life and family roles. Overwhelm was frequently named.

Commentary on the emotional experience was largely present in YP + and YP- interviews, as well as P +. Participants described a range of difficult emotions, including shame (P + BG0162), bitterness (YP + YG0078), frustration (YP + BN0074), and anger (YP + YG0082), with these feelings linked to a deeper sense of meaninglessness:

“I needed to process the idea that I was going to live without my father…. Oh my God, I harmed myself to the extent that I felt like living was meaningless” (YP + YG 081).

This lack of meaning was apparent in cases where a young person had died by suicide:

“She chose death rather than a life without meaning.” (P + YG 84)

A sense of worthlessness was common, including feeling as if they did “not exist” and were not present (YP + BG 0148*).* HCP and YP + mostly mentioned this, with links to not having a role or function in life, particularly in family. This reflected the implicit judgements made about a person’s worth in relation to social norms, their own expectations, and the importance of having and supporting family:

“But I reflect on what I've been through and feel useless, especially when I consider my age and the fact that I've given birth"…YP + YN 0059

A lack of hope was described in more intra-personal terms, focusing on not having “any future hope” (YP + YG0078). At its extreme, people described entrapment. Entrapment was strongly associated with the intention to die.

“When I decided to commit suicide [sic], it was because I felt like I would spend my whole life listening to him [Father]” (YP + BN 0090).

Isolation was a cause and outcome of distress. Distress was hidden from others: “their peers are often unaware of their intentions; only the individual knows.” (HCP G). This amplified “loneliness” (YP-YG0071). Many participants stories included a sense of isolation from others, for example being mocked by others at school and not finding a friendship group (YP + YG0082).

### Theme 3: Functions and characteristics of self-harm

Described methods used to harm oneself or end their own life were varied, including cutting, poisoning (with drugs, household/agriculture chemicals, or extreme use of alcohol), hanging, jumping into river/lakes, starving oneself, and burning oneself (predominantly without intention to die). Self-harm served intrapersonal and interpersonal functions, including emotional regulation, communication of distress, punishment, and efforts to regain a sense of control.

For many young people, self-harm was to find relief from overwhelming emotions. Participants spoke about how the build-up of emotional pain led them to see self-harm as the only outlet. A sense of no escape from emotional suffering often made self-harm with the intention to die feel like the only solution, to end unbearable distress:

“I felt like it was going to help me find rest, that’s what I thought. I believed I would have fewer problems when I died.” (YP + BG0148)

Shame also played a powerful role in self-harming behaviour. Fear of social humiliation pushed individuals towards suicide as a way to avoid disgrace:

“What I think she was thinking is that instead of being ashamed in front of others [with unplanned pregnancy], it is better to commit suicide to avoid being seen.” (P + YN38)

Not all self-harm had the intention to die. Many participants discussed self-harm as a coping mechanism to manage overwhelming emotions, to release emotional pain, deal with feelings of self-hatred, or cope with loss. Indeed, for some suicidal ideation itself provided a sense of relief:

“They [suicidal thoughts] were helpful in that I would be thinking that I’m about to die, and rest, and the sufferings in the world will end.” (YP + YN0037)

Healthcare providers also recognised this function, describing self-harm as a way for young people to manage distress related to poverty, trauma, or mental health struggles. They also expressed concerns about perceptions of positive functions of these behaviours, seeing them as “detrimental”, “not a solution”, and potentially physically harmful in the long-term.

Interpersonal functions were also clear, particularly communication of pain to others, for example “It was a way to show the weight of my anguish.” (HCP Go). It could signify a need for help:

“I think the person who decided to harm him or herself had something happen to them. I understand it as a person who faces hardships and needs someone to sympathise with them.” (YP + YN0052)

Other relational purposes included a belief that dying by suicide would ease the burden on their families. A parent recounted, “He said maybe if I am no longer on the earth, you will be at peace.” (P + YN 0037). Conversely, it could be used to punish others in the family:

“The girl told me, she has no problem, we only wanted you [stepmother] to get arrested.” (P + BG0115)

### Theme 4: maintenance and cessation of self-harm

#### Barriers to cessation

HCPs highlighted the lack of awareness among youth regarding the long-term harm of self-harm, emphasising the need for better education on its consequences and the importance of early intervention. They felt that this would support young people to stop repeated self-harm. A YP + reflected on how hard it was to stop because it helped them:

“I would have quit. But because I liked it, I would repeat, but then go back” (YP + BG 0132).

Many participants were unaware of available resources: “You don't even know that the thing exists.” (YP + BG 0132). Even when aware, some were too emotionally overwhelmed to seek help:

“I didn’t feel like I needed help. I felt that I had no reason to live and had no reason to seek help.” (YP + BG 0148).

A lack of social support was linked to ongoing self-harm. This was mentioned predominately by YP +, owing to their direct personal experience of this. For some, this was due to the absence of a supportive person (YP + BN 0099), and others found it too difficult to describe their difficulties to another person, or simply felt there was no-one to help them.

### Pathways to cessation

Predominately seen in the YP +, owing to their direct experience, participants explained how self-harm may stop. Many described using cognitive strategies to cope with difficult situations, such as finding positive reframes and moments, or focusing on a sense of purpose. Strategies such as distraction using entertainment on their phone (YP + YG0078), or exercise such as running (YP + BN0074) were also described. School was seen as helpful in stopping self-harm, although it was unclear if this was due to social support or distraction, or other reason such as how school may provide a sense of hope for a positive future.

Community and peer support were clearly named as helpful:

“and community peer support [an organisation called] OPROMAMER has helped me” (YP + BG 0148)

Peer support provided the experience of being listened to, social support of people who “check on them” (P + BG0160), and guidance and advice. Some participants talked about having specific people to confide in, to “go tell [her] how I feel” (YP + BG0132), specifically linking this in the interviews to being able to stop self-harm or manage suicidal thoughts. Praying, spiritual practice, and confiding in God were also seen as supportive.

When participants did talk about support received from health-care providers, this focused on feeling cared for and having an opportunity to talk, with positive effect.

### Theme 5: duality of community perceptions and responses—harmful and helpful

The community represented a site of judgement, rejection and risk, as well as support and recovery.

Three subthemes were developed: Stigma, silence and harmful judgement; ambiguous support; and containment, care and connection.

#### Stigma, silence and harmful judgement

Social and community perceptions played a part in defining some of the triggers/reasons for self-harm. The experience of stigma based on community perceptions was a causal and maintaining factor. Participants from all groups discussed rejection and gossip within the community, especially towards those facing unplanned pregnancies or gang involvement, which were socially defined failings. Importantly, in some YP + talk, this community responses were direct causes for wanting to kill themselves. Stigma from community, in relation to the young person’s family members, was a direct causal factor for some young people:

*"People want to blame us for my father’s behaviours who doesn’t live with other people well, and some people bad mouth me saying: look at the son of the other [man] who is always insulting us.” (YP* + *YN 001).*

The response from some in her community was hurtful to this young person, however she also explained she had supportive friends who helped her manage the distress, highlighting the dual impacts of responses from others.

Beliefs about self-harm held by the community, and therefore also by some of participants, included a link to spiritual causes. These sat alongside other perceptions of cause, an element in a complex, multidimensional set of beliefs. Participants described their own and other community members perceptions linking to “bewitchment”. One described how she was seen as *Inkunguzi* – a bad omen, someone who brings misfortune to others because of her self-harm (YP + YG 081). Others spoke about “demons”: “They say it is a demon that attacks him/her” (P-YN49). This linked to high levels of social stigma and potentially fear, and a lasting consequence of a person who has self-harmed being excluded.

Self-harm and death by suicide were described as unacceptable, unchristian, uneducated, criminal, satanic, cursed, and linked to bad parenting. Parents of young people who had self-harmed (P +) frequently described this social judgment. There was also a community belief linking self-harm to unplanned pregnancy, drugs, and alcohol:

*“Living a bad life [are the] reasons these young people try to commit suicide, those temptations we talked about [referring to drugs, alcohol, sex, and engagement in delinquent behaviours]” (YP-BN0063).*

Whilst these factors represented just one narrative of causes, this perceived link meant that self-harm was de facto perceived as “bad” and would be “punished” (HCP C) or rejected, owing to the “disgrace” (HCP, K). This then had social consequences of being isolated.

These perceptions of self-harm as unacceptable inadvertently maintained the behaviours, maintaining the build-up of emotions linked to lack of hope or belonging. The consequences of self-harm were “always feeling lonely and thinking that society would not accept them as someone who has done unthinkable things” (HCP C). This led to a sense of self and isolation, which may itself be a cause for the behaviours.

Not only were self-harm and the young person doing this seen negatively, so were their parents, who were seen as a causal factor. Health care professionals described how the community’s perceptions of mental health could lead to bullying, mocking, and shame on the family overall, with an indelible label, “which itself has a negative impact” (HCP G). Shame from being known as someone who has self-harmed, or has a child who has, then creates a vicious cycle, whereby they shame inhibits help-seeking for the young person and community engagement and support for the parent:

“They [the young person] feel that if they seek help, people might laugh at them…they feel embarrassed and scared to seek help” (YP-BG0121).

This perception of the parents then fed back to the parent–child interaction, and the child’s perception of themselves. In response to social stigma, one parent described how.

“I asked him [son] not to make me ashamed in society and why he couldn’t be like other good boys” (P + YG87).

This response may deepen the negative self-perceptions and emotional build up, unwittingly creating a vicious cycle.

Community responses were interwoven into the parents’ experience, where neighbours became involved in talking to the family (e.g. P + NG0015), as did village leaders (P + 75). These discussions ranged in supportive through advice to the parent or young person (P + 83), to more punitive: “They scolded him verbally” (P + YG87). Indeed, in many cases, input from the community focused on the idea that self-harm was bad and would lead to bad consequences in this life, and in any afterlife.

The parents and health-care professionals viewed such support as useful. In our analysis we noted how, as a group of psychologists with training either in a UK model or informed by dominant “Western” theories, that we held the assumption this would be harmful. Mindful of this, we noted YP + did describe this negatively, when responding to a prompt around things that had been unhelpful for them:

“Meeting someone who advises me, people giving me advice, telling me that despite the problems at home, … I should keep going,… Those people, even now, I don't like them” (YP + BN 0074)

#### Containment, care and connection

The community played a helpful role in many instances, from friends, parents, wider family, and neighbours. Institutions such as the church and school had been experienced as supportive:

“ever since I attempted suicide, I met with many people, either church members who talked to me and other different people, I immediately realized that the illness I have should heal” YP + BN 0090

Community leaders and neighbours had also provided life-saving support in several cases, for example.

“They found me hanging myself with a belt and prevented me from doing it” YP + YG 0078

This support took various formats: being told this was an illness requiring hospital treatment (YP + YG0078); discussions experienced as feeling cared for (YP + YG0082); support to find a sense of acceptance (YP + YN 0034); a sense of hope (YP + BN0090); and practical advice to use coping techniques such as distraction (YP + YN0035). Peer support interventions, such as that provided by the charity “OPROMAMER”, a mental health peer support organisation, provided a sense of universality:

“by listening to others' testimonies, I realized I was not the only one who had encountered such things.”(YP + YN0034)

Distortion of family structures, in some part link to the 1994 genocide, were highlighted by some participants.

“I have never seen my uncle. My grandma has come only once, and our only father has left us. Grandpa is in Uganda, and we are alone” (YP + YN 0052).

HCPs and parents reflected on how such changes reduced traditional support systems:

*“In the past, children used to go to their grandfathers… now do they exist?”* (HCP KG).

*“Family lives in a scattered system, with no child visiting uncles or grandparents.”* (P-YN45). In some cases, then layers of community support were missing, for the young person and parents. However, opportunities for family and community support were still present:

“It is a cultural practice where the youth come together with the elderly people for conversation, and that’s where they can receive advice that can support them.” (HCP C).

The content of the teachings, as has been demonstrated, has the potential for both help and harm.

## Discussion

This study aimed to explore the experiences and perceptions of self-harm in young people in Rwanda, through thematic analysis of interviews with young people and parents with and without lived experience of youth self-harm, and health-care professionals. Our five themes illuminate how participants perceived and made sense of youth self-harm. Our analysis revealed patterns of lived experience in which young people described the build-up of distress, their efforts to regulate feelings or communicate through self-harm, and the ways they thought about stopping or continuing. The analysis highlights commonalities and differences in perspectives among young people, parents, and healthcare professionals, and shows how these perspectives were embedded in psychological, social, cultural, and community contexts. Community responses were described as cutting across all themes, influencing both how self-harm was understood and how it was managed.

In the discussion, the concept of self-harm and how it is influenced by multiple levels of analysis is considered. We then explore the drivers of distress, before highlighting theoretical insights and relevance to policy. Areas for intervention are described throughout and summarised in Table 4. Finally, we reflect on the study’s reflexivity, strengths, limitations, and directions for future research.

**Table 4:** Recommended intervention areas and rationale from the data

Area for intervention	Rationale
Increase support for families to manage conflict and reduce abuse, as well as increasing emotional expression. For example, community-based parenting support embedded into Umugoroba w'Ababyeyi and church programmes	Participants specifically described abuse and challenging family relationships as a reason for self-harm/suicidality. Young people particularly described feelings of rejection and lack of love as contributing to the build-up of emotions that were part of why they engaged in self-harm/suicide. This may include support from community and religious leaders, with training for them as required
Continue and develop the offers of peer-support organisations, for example, support and expand initiatives like OPROMAMER and youth peer groups	Participants talked about a lack of support, isolation, and a need to feel cared about. Peer support organisations, such as OPROMAMER, may directly address this need, in lieu of this being provided in an informal manner
Cross-sector policy and initiatives are essential to self-harm reduction. For example, poverty alleviation and school access interventions should be seen as crucial programs to reduce self-harm	The impact of poverty and, often by extension, lack of access to school on young people’s emotional life especially after unplanned pregnancy, sense of value and sense of future were significant. These issues presented as underlying reasons that drive young people to self-harm and suicidality
Increase range of coping mechanisms and ways to manage distressing emotions, for example culturally relevant emotional regulation techniques like distraction, exercise, spending time with trusted others	Young people did describe the function of self-harm and suicidality as linked to managing or regulating their emotional distress. Exploring how to offer other strategies that are linked to culture, including some of those named by participants (distraction, exercise, time with others) may then offer alternative behaviours
Community discussion and development of appropriate initiatives to increase availability of emotional support, for example, village based safe spaces, community mental health initiatives	Participants talked about the need for young people to have a place and someone safe to talk to, to express their emotions to and help to avoid a sense of isolation and overwhelm. This may be achieved through community and religious leaders
Community discussion relating to how young people experience distress, self-harm and suicidality. For example, this could include “community conversations” or participatory workshops, using drama to share youth voices	There were key differences in how young people talked about their experiences and the viewpoints of parents in relation to the importance of trigger factors, emotional experience, support desired, and impact of community beliefs. Sharing the young people’s own descriptions and conceptualisations could lead to greater understanding across the community. This may be achieved through community and religious leaders

### Understanding youth self-harm in context

The distinction between self-harm with the intention to die, or not, was evidenced in some data. For example, the link between entrapment and a sense that life was not worth living was strongly linked to intention to die. However, the distinction was unclear and complex, sometimes with a lack of clarity in participants’ talk as to whether they were referring to fatal or non-fatal acts. These data suggest that binary distinction around intent to die can mischaracterize the experience of despair among some youth as well as the focus and function of self-harm as relief from distress. Intention towards death may be fluid and is not consistently foregrounded as a focal point in the experiences.

Our themes weave across different levels of analysis, including lived experience of emotion, relational dynamics, structural context (e.g., poverty, access to education), community responses, and higher levels cultural beliefs. This in part reflects the data collection tools. HCPs’ accounts highlighted structural context relating to awareness of services, and their observations of how community stigma had impacted help-seeking. This supports the lived experiences of young people and parents. Descriptions about emotional distress, for example, focused on individual level (the actual emotional experience), the aspects that are based on relationships with others (e.g. distress because am unable to support others YP + BG081), a structural contextual level (e.g. concerns for the future owing to difficulties accessing education), and community level (e.g. how others respond to self-harm, be that supportive or stigmatising). Of significance for theory, this shows the importance of models of self-harm to consider multiple levels. Furthermore, this reinforces that multi-level interventions are essential, owing to this inter-twined nature of different levels.

Whilst in many cases there were similarities in the talk of the different participant groups, there were differences in understandings relating to the way in which peer relationships were important, the range and extent of the emotional and psychological distress of young people and their desire to have emotional support. As community beliefs and responses influenced the genesis and maintenance of self-harm, addressing differences in understandings between community, parents, and young people is core to appropriately support young people. Psychoeducation during community discussions such as Umuganda (community service), Umugoroba wababyeyi (Parents’ evening), and others based firmly on the voices of the young people, may begin to bridge this difference in understanding and avoids giving epistemic privilege to content from the Global North.

### Family, structural and cultural drivers

Participants consistently described family relationships as central to their accounts of self-harm, particularly experiences of parental rejection, neglect, and lack of emotional support, which they linked to young people’s sense of self-worth and emotional wellbeing. Parental rejection and lack of emotional support contributed to the build-up of distressing emotions. Rejection was also seen as a key factor in research in Nigeria [6], underscoring the importance of belonging, as a fundamental human need [46] but also as particularly important in cultures with a greater focus on community and collectivism [47]. The concept of “*Ubumuntu*” (“*Ubuntu*” in some southern African languages) meaning “I am because we are”, reflects this emphasis on relational interdependence and shared responsibility [48]. Indeed, when discussing what helped young people to stop self-harm, social connection was key with some using Kinyarwanda proverbs such as “Inkingi imwe ntigera inzu (one pillar cannot support a house)” to reflect the significance of collective support to overcome distress. This could be family as a safe place to talk, peer support through school and friends, and formal peer-support organisations. Such protective factors and coping strategies are important to draw attention to: positive, loving relationships, a sense of hope and purpose, and coping mechanisms such as focusing on the positives or finding meaning in life were frequently cited. While these factors are not novel, they have received limited attention in sub-Saharan African contexts [21].

The practical implications for interventions is to focus not only on reducing family conflict and abuse, as seen in existing parenting programmes in Rwanda [49], but on increasing moments of expression of love, and opportunities to discuss emotions. Furthermore, peer support initiatives play a vital role where family support is not achievable through providing a sense of belonging and mitigating loneliness. Interventions must be context suitable, and could be something akin to social interventions to create “one good adult” for each young person in their lives to be able to talk to, potentially working with communities to understand the importance of this and explore training options, as in Ireland [50].

Poverty was a pervasive factor contributing to self-harm, mentioned by all participant groups and supported by significant literature [5, 14]. It multiplied and amplified other stressors, leading to increased overwhelm and reduced capability to escape many situations, intensifying entrapment. Access to school was a driver for self-harm and linked to poverty. Poverty reduction and school access interventions and policies should be seen as part of mental health and suicide prevention work.

Beliefs held within the community, relating to spirituality, religious beliefs, and moral judgements, were critical and often deepened the sense of shame and distress for the young people. Such beliefs are seen in a variety of contexts and are often held in combination with other beliefs [51]. The impact of community ideas about self-harm as “madness” impact people globally [e.g., 52], therefore this issue is specific in content but not consequence. The challenge for future intervention is to explore how to manage the sometimes-harmful impact of these ideas on young people, and how such beliefs such as “Indwara zose si izo kwa muganga (Not all illness are meant to be treated by a doctor)” may integrate with the support strategies that young people described valuing. This is relevant beyond Rwanda to settings where similar ideas may be present in communities.

Seen through the experiences shared in the interviews, community beliefs intersected with broader patterns of meaning, through which young people made sense of self-harm. Participants spoke of no rest and the search for rest to capture relentless suffering, with resonance in both everyday hardship and potentially Christian notions of peace. Experiences of entrapment reflected limited choice within family hierarchies and poverty. The idea of having or not having ‘a life’ was tied to disrupted trajectories of schooling and work, giving rise to feelings of meaninglessness and uselessness. The impossibility of invisibility in tightly connected communities heightened shame when distress was visible and judged. Finally, self-harm was sometimes framed in terms of badness or demonic attack, drawing on moral-spiritual vocabularies that could deepen stigma but also prompt care. These patterns situate self-harm within Rwanda’s cultural narratives of worth, belonging, and honour, connecting individual suffering with wider moral, economic, and social worlds.

### Extending theory

Existing psychological models of self-harm and suicide do incorporate relational, emotional, and contextual factors. Our findings call for a more explicit consideration of structural and cultural mediators within these theoretical frameworks, particularly when applied across diverse socio-cultural settings.

Our findings offer support and important extensions to existing psychological models of self-harm. In line with the Emotional Regulation theory of self-harm [53], a function of self-harm was to clearly manage difficult feelings. The Integrated Motivational-Volitional (IMV) model and Emotional Regulation models both suggest that self-harm emerges from processes of emotional overwhelm, entrapment, and cognitive constriction [54]. Entrapment was clear in the emotional experiences of young people. IMV motivational moderators (factors that impact the move to suicidal ideation) such as social responses, were particularly evident: family and community rejection, and stigmatising attitudes, appeared to directly influence emotional entrapment.

The relevance of this broad socio-cultural moderators of motivation are culturally specific, and therefore must be considered in relation to each context. Structural inequalities, especially poverty, exacerbated emotional vulnerability by increasing experiences of hopelessness and worthlessness. The link between the social motivational moderators and emotional experiences was strong. Feelings of self-hate and worthlessness were often linked to a failure to meet social expectations, or to contribute meaningfully to others, an important dimension in collectivist societies, where self-worth is socially anchored [55]. Across many cultures, emotional expression may not be socially endorsed and can be framed as immaturity or indulgence [56, 57]. Such cultural norms likely amplify emotional suppression and distress, shaping self-harm trajectories.

The Interpersonal theory of suicide highlights its relational functions [58]. Thwarted belongingness and perceived burdensome were clearly evidenced in our findings. Functions of punishment or revenge were also seen and have been documented in young people, linked to perceptions of neglect or harm from others [59]. Economic responsibility and family expectations were particularly important here. In this context, belongingness was not purely interpersonal, but also related to broader community status and cultural definitions of worth.

The interpersonal theory of suicide includes the idea of “acquired capability”, relating to learning to overcome a fear of death and pain, necessary to then kill oneself [58]. This is thought to be typically developed through repeated exposure to trauma and pain. Our findings highlight this is also linked to cultural and social norms emphasising silent endurance of suffering and emotional restraint, leading young people to attempt to suppress. This may be linked to acquiring the capability of self-harm/suicide: the barriers to this are lower owing to cultural messages about hiding distress, meaning help is not sought. The theory must be extended to include consideration of community structures and cultural beliefs as mediators of interpersonal risk.

### Transferability of findings

The majority of young people’s deaths by suicide occur in low-middle income countries [1], therefore research in these settings is key. Self-harm is rooted in culture and content, demanding models that are sensitive to context [60, 61]. Whilst each context is unique, issues identified in this study including poverty, access to schooling, and family structures, are shared to some extent in other regions of East Africa and the broader Global South. Further research is needed to explore how poverty and disempowerment contribute to entrapment and self-harm risk, and how interventions can be designed for contexts where interdependence, familial obligations, and collective identity are central. Although caution must be applied, and local adaptations considered, these findings offer insights that may inform understanding of self-harm pathways across similar cultural and socio-economic settings, particularly within East Africa.

### Policy implications

These findings highlight the need for integrated, cross-sectoral policy responses to youth self-harm. Self-harm was not solely a mental health issue, but intersected with education, economic development, family support, adolescent pregnancy, and substance use. Addressing youth self-harm requires coordinated strategies that extend beyond health services, including policies improving education access, poverty reduction, supporting young parents, strengthening community mental health capacity, and tackling substance misuse among young people. Interventions must also account for the cultural and structural dimensions of distress, such as community stigma, spiritual beliefs, and disrupted family networks. Although this study focused on Rwanda, the social-structural drivers identified are common across many low- and middle-income and post-conflict settings, suggesting broader policy relevance. Developing culturally sensitive, multi-sectoral approaches is critical for effective prevention and support. This may be challenging in resource-limited settings. Using existing infrastructure to work cross-sector may be one appropriate way forward.

### Reflexivity and cross-cultural research practice

We conducted research on youth self-harm across linguistic and cultural contexts, with an international team. This required a commitment to reflexivity, transparency, and epistemic justice [62]. Each person brought diverse perspectives that shaped both analysis and interpretation. Discrepancies in assumptions, for example, the role of emotional expression or the impact of poverty, were surfaced in our in-person discussions and were treated as productive tensions, prompting iterative re-engagement with Kinyarwanda transcripts and prioritisation of local analytic voice [63]. Time spent building rapport and psychological safety as a research team was essential to support this. Responding to work to decolonise global mental health research [64], we avoided imposing external frameworks during analysis, instead trying to privilege the knowledge from our participants. Validation/”member checking” revealed power asymmetries when professionals dominated discussion, leading us to adapt our method to a participatory, drama-based approach that enabled their input through a familiar and perhaps safer mode of interaction [65]. Analytical transparency was addressed through team discussions and shared reflexive notes. Ethical reflexivity included deliberate strategies to avoid gratuitous trauma and voyeurism when quoting participants [66]. This approach supported rigour, transparency, and openness, operationalised through creative, contextually grounded practices, to work towards ethically robust and culturally responsive psychological research in a Global South settings. Further details of our methodological adaptations and reflexive processes are provided in Additional File 3.

### Strengths, limitations and future directions

This study's strengths included the sampling of participants from diverse backgrounds, including different experiences of youth self-harm and a mix of rural and urban areas, allowing for a range of perspectives. The analysis process, involving a culturally diverse research team, supported reflexivity and helped interrogate potential biases. Rigour was further enhanced through member checking with participants and expert input into theme development.

Limitations included the challenge of disentangling experiences of self-harm with and without suicidal intent. While this ambiguity may reflect participants’ real-world conceptualisations, it highlighted the need for further exploration and possibly additional data collection. Whilst no participant was excluded explicitly on the basis of gender, we did not specifically seek to include transgender or gender minority youth in our sample, meaning their voices are not represented in our work and future research should explicitly seek to recruit this group. It may be seen that working with government actors could have impacted on our recruitment process, however in this context, it was essential to have support and acceptance from village leaders to be able to approach members of the community, respecting local structures and community norms. Their gatekeeper role, including permissions, introductions to community members, and the trust built in the community by working through the existing structures supported recruitment, as seen in other studies [67]. At no point were any members of the government made aware by the research team of who had volunteered or not, and the research teams experience of working in this context led discussions with leaders around respecting people’s right to choose to take part or not. Our sample was self-selected, with many participants already accessing support services, and there was an under-representation of young men, particularly those without self-harm experience (31% male overall, with only one YP- male from a rural region). These factors may have influenced the range of perspectives captured.

We intend to further analyse these data to explore in depth additional issues such as parents’ distress at their young person’s self-harm, healthcare providers’ perceived skills and knowledge in responding to self-harm and suicide and a detailed comparison of different participant groups’ viewpoints, organised by a theoretical framework such as the common-sense model [68]. The research team is composed of Clinical Psychologists and early career research psychologists. This brings bias associated with therapeutic training, however this was somewhat mitigated by the range of positions held regarding orientation towards medical, psychological, or social models of mental health and the range of cultural backgrounds across the team.

Further research should target young men specifically, explore barriers to their participation, and address the ongoing challenge of differentiating self-harm with and without intent to die. Longitudinal, quantitative, and epidemiological research is needed to track the evolution of risk and protective factors and establish self-harm prevalence across different groups. Intervention development should be informed by the contextual findings presented here and address the potential targets outlined in Table 4.

## Conclusions

This study aimed to explore how young people, parents, and healthcare professionals in Rwanda perceived and made sense of youth self-harm, and how these experiences were embedded in family, community, structural, and cultural contexts. The findings highlight the non-duality of self-harm, with participants describing it in ways that crossed distinctions between suicidal and non-suicidal intent. Family conflict, poverty, and disrupted educational and social trajectories were consistently described as shaping young people’s distress, while cultural and community influences, including stigma, visibility, and spiritual-moral interpretations, were seen as central to how self-harm was understood and responded to. Community responses were described as cutting across all themes, suggesting pathways by which stigma or support could intensify or relieve young people’s suffering. Together, these findings underscore the need for multi-level approaches that recognise the interplay of individual, relational, structural, and cultural factors, and they align with and extend existing theoretical models by showing how cultural narratives of worth, belonging, and honour frame the meanings attached to self-harm in Rwanda. Our findings suggest the value of expanding suicide theories to incorporate social ecologies, cultural moral economies, and historical trauma as fundamental to understanding risk and resilience. Effective prevention efforts should be contextually grounded, culturally sensitive, and multi-sectoral in order to reflect the complex realities facing young people.

## Supplementary Information

Additional file 1.

Additional file 2.

Additional file 3.

## Data Availability

The datasets used during the current study are available from the corresponding author on reasonable request. To preserve the anonymity of participants, not all raw data will be made publicly available.

## References

[1] WHO. Suicide in the world: global health estimates. Accessed online June 2023 https://www.who.int/publications/i/item/9789240026643: World Health Organization; 2019.

[2] UN. United Nations: Transforming our world: The 2030 agenda for sustainable development. UN General Assembly. Available online https://sustainabledevelopment.un.org/content/documents/21252030%20Agenda%20for%20Sustainable%20Development%20web.pdf: Tech. Rep. 1; 2015.

[3] Knipe D, Padmanathan P, Newton-Howes G, Chan LF, Kapur N. Suicide and self-harm. Lancet. 2022;399(10338):1903–16. 35512727 10.1016/S0140-6736(22)00173-8

[4] NICE. Self-harm: assessment, management and preventing recurrence [NG225]. Accessed online https://www.nice.org.uk/guidance/ng225 October 2022: National Institute for Clinical Excellence; 2022. 36595613

[5] Moran P, Chandler A, Dudgeon P, Kirtley OJ, Knipe D, Pirkis J, et al. The Lancet commission on self-harm. Lancet. 2024;404(10461):1445–92. 39395434 10.1016/S0140-6736(24)01121-8

[6] Jidong DE, Ike TJ, Husain N, Francis C, Husain MO, Mwankon SB, et al. Perspectives on self-harm and suicidal ideation in Nigeria: a mixed-methods study of patients, family caregivers, clinicians, and the public. Arch Suicide Res. 2024;28(4):1417–31. 38363148 10.1080/13811118.2024.2314520

[7] Muehlenkamp JJ, Kerr PL. Untangling a complex web: how non-suicidal self-injury and suicide attempts differ. Prev Res. 2010;17(1):8–10.20835367

[8] Kapur N, Cooper J, O’Connor RC, Hawton K. Non-suicidal self-injury v. attempted suicide: new diagnosis or false dichotomy? Br J Psychiatry. 2013;202(5):326–8. 23637107 10.1192/bjp.bp.112.116111

[9] Wachter Morris CA, Wester KL. Functions and prevalence of self-directed violence in adolescence. J Child Adolesc Couns. 2020;6(2):110–23.

[10] De Leo D, Goodfellow B, Silverman M, Berman A, Mann J, Arensman E, et al. International study of definitions of English-language terms for suicidal behaviours: a survey exploring preferred terminology. BMJ Open. 2021;11(2):e043409. 33563622 10.1136/bmjopen-2020-043409 PMC7875264

[11] Stänicke LI, Haavind H, Gullestad SE. How do young people understand their own self-harm? A meta-synthesis of adolescents’ subjective experience of self-harm. Adolesc Res Rev. 2018;3(2):173–91.

[12] Knipe D, Metcalfe C, Hawton K, Pearson M, Dawson A, Jayamanne S, et al. Risk of suicide and repeat self-harm after hospital attendance for non-fatal self-harm in Sri Lanka: a cohort study. Lancet Psychiatry. 2019;6(8):659–66. 31272912 10.1016/S2215-0366(19)30214-7 PMC6639451

[13] World Economic Forum. A Global Framework for Youth Mental Health: Investing in Future Mental Capital for Individuals, Communities and Economies. In: Orygen, editor. Accessed online July 2021 http://www3.weforum.org/docs/WEF_Youth_Mental_Health_2020.pdf. 2020.

[14] Bantjes J, Iemmi V, Coast E, Channer K, Leone T, McDaid D, et al. Poverty and suicide research in low- and middle-income countries: systematic mapping of literature published in English and a proposed research agenda. Glob Ment Health. 2016;3:e32. 10.1017/gmh.2016.27 PMC5454768 28596900

[15] Osafo J, Hjelmeland H, Akotia C, Knizek B. Social injury: an interpretative phenomenological analysis of the attitudes towards suicide of lay persons in Ghana. Int J Qual Stud Health Well-being. 2011;6(4):8708. 10.3402/qhw.v6i4.8708 PMC3209819 22065981

[16] Aggarwal S, Borschmann R, Patton GC. Tackling stigma in self-harm and suicide in the young. Lancet Public Health. 2021;6(1):e6–7. 33417848 10.1016/S2468-2667(20)30259-0 PMC7611270

[17] Asiimwe R, Dwanyen L, Subramaniam S, Kasujja R, Blow AJ. Training of interventionists and cultural adaptation procedures: a systematic review of culturally adapted evidence-based parenting programs in Africa. Fam Process. 2023;62(1):160–81. 35570371 10.1111/famp.12780

[18] Mabrouk A, Mbithi G, Chongwo E, Too E, Sarki A, Namuguzi M, et al. Mental health interventions for adolescents in sub-Saharan Africa: A scoping review. Frontiers in Psychiatry. 2022; Volume 13 - 2022. 10.3389/fpsyt.2022.937723 PMC9429610 36061286

[19] Theron L. Championing the resilience of sub-Saharan adolescents: pointers for psychologists. S Afr J Psychol. 2019;49(3):325–36.

[20] Quarshie ENB, Waterman MG, House AO. Adolescent self-harm in Ghana: a qualitative interview-based study of first-hand accounts. BMC Psychiatry. 2020;20(1):275. 32487040 10.1186/s12888-020-02599-9 PMC7268665

[21] Quarshie ENB, Waterman MG, House AO. Self-harm with suicidal and non-suicidal intent in young people in sub-Saharan Africa: a systematic review. BMC Psychiatry. 2020;20(1):234. 32408896 10.1186/s12888-020-02587-z PMC7222461

[22] Zulu JM, Budhwani H, Wang B, Menon A, Kim D, Zulu M, et al. Living a private lie: intersectional stigma, depression and suicidal thoughts for selected young key populations living with HIV in Zambia. BMC Public Health. 2024;24:1937. 39030515 10.1186/s12889-024-19278-z PMC11264630

[23] Ngwane VE, van Der Wath AE. The psychosocial needs of parents of adolescents who attempt suicide. J Psychol Afr. 2019;29(4):375–82.

[24] Kritzinger AM. Investigations into adolescent non-fatal suicidal behaviour at a Gauteng public hospital: Patient and staff experiences.DP - 2024. Dissertation Abstracts International: Section B: The Sciences and Engineering. 2024;85(6):No Pagination Specified.

[25] Index Mundi. Rwanda Age Structure. Accessed online Aug 2021. 2020 https://www.indexmundi.com/rwanda/age_structure.html.

[26] Muwonge J, Umubyeyi A, Rugema L, Krantz G. Suicidal behaviour and clinical correlates in young adults in Rwanda: a population-based, cross-sectional study. J Glob Health Rep. 2019;3:e2019080.

[27] Ng LC, Kirk CM, Kanyanganzi F, Fawzi MC, Sezibera V, Shema E, et al. Risk and protective factors for suicidal ideation and behaviour in Rwandan children. Br J Psychiatry. 2015;207(3):262–8. 26045350 10.1192/bjp.bp.114.154591 PMC4555444

[28] Mutuyimana C, Cassady C, Sezibera V, Nsabimana E. Prevalence and correlates of depression among rural and urban Rwandan mothers and their daughters 26 years after the 1994 genocide against the Tutsi. Eur J Psychotraumatol. 2021;12(1):2005345. 34900124 10.1080/20008198.2021.2005345 PMC8654415

[29] Perroud N, Rutembesa E, Paoloni-Giacobino A, Mutabaruka J, Mutesa L, Stenz L, et al. The Tutsi genocide and transgenerational transmission of maternal stress: epigenetics and biology of the HPA axis. World J Biol Psychiatry. 2014;15(4):334–45. 24690014 10.3109/15622975.2013.866693

[30] Mutuyimana C, Sezibera V, Nsabimana E, Mugabo L, Cassady C, Musanabaganwa C, et al. PTSD prevalence among resident mothers and their offspring in Rwanda 25 years after the 1994 genocide against the Tutsi. BMC Psychol. 2019;7(1):84. 31856892 10.1186/s40359-019-0362-4 PMC6923965

[31] Jensen SKG, Sezibera V, Murray SM, Brennan RT, Betancourt TS. Intergenerational impacts of trauma and hardship through parenting. J Child Psychol Psychiatry. 2021;62(8):989–99. 33284991 10.1111/jcpp.13359

[32] Roth M, Neuner F, Elbert T. Transgenerational consequences of PTSD: risk factors for the mental health of children whose mothers have been exposed to the Rwandan genocide. Int J Ment Health Syst. 2014;8(1):12. 24690436 10.1186/1752-4458-8-12 PMC3978019

[33] Thippaiah SM, Nanjappa MS, Gude JG, Voyiaziakis E, Patwa S, Birur B, et al. Non-suicidal self-injury in developing countries: a review. Int J Soc Psychiatry. 2021;67(5):472–82. 32715834 10.1177/0020764020943627

[34] Martin F, Ferrey A, Hobbs L, Lascelles K, van Even S, Oliver T. Understanding the impact of children’s and young people’s self-harm on parental well-being: a systematic literature review of qualitative and quantitative findings. Child Adolesc Ment Health. 2024. 10.1111/camh.12692. 38362819 10.1111/camh.12692

[35] Nguyen HTL, Nakamura K, Seino K, Vo VT. Relationships among cyberbullying, parental attitudes, self-harm and suicidal behavior among adolescents: results from a school-based survey in Vietnam. BMC Public Health. 2020;20(1):476. 32276608 10.1186/s12889-020-08500-3 PMC7146902

[36] Rugema L, Krantz G, Mogren I, Ntaganira J, Persson M. “A constant struggle to receive mental health care”: health care professionals’ acquired experience of barriers to mental health care services in Rwanda. BMC Psychiatry. 2015;15(1):314. 26672596 10.1186/s12888-015-0699-z PMC4682265

[37] Mukashema I, Mullet E. Unconditional forgiveness, reconciliation sentiment, and mental health among victims of genocide in Rwanda. Soc Indic Res. 2013;113(1):121–32.

[38] Braun V, Clarke V. Thematic analysis: A practical guide. Sage publications; 2021.

[39] Sundler AJ, Lindberg E, Nilsson C, Palmér L. Qualitative thematic analysis based on descriptive phenomenology. Nurs Open. 2019;6(3):733–9. 31367394 10.1002/nop2.275 PMC6650661

[40] Tong A, Sainsbury P, Craig J. Consolidated criteria for reporting qualitative research (COREQ): a 32-item checklist for interviews and focus groups. Int J Qual Health Care. 2007;19(6):349–57. 17872937 10.1093/intqhc/mzm042

[41] Squires A. Methodological challenges in cross-language qualitative research: a research review. Int J Nurs Stud. 2009;46(2):277–87. 18789799 10.1016/j.ijnurstu.2008.08.006 PMC2784094

[42] van Nes F, Abma T, Jonsson H, Deeg D. Language differences in qualitative research: is meaning lost in translation? Eur J Ageing. 2010;7:313–6. 21212820 10.1007/s10433-010-0168-y PMC2995873

[43] Dhakal K. NVivo. J Med Libr Assoc. 2022;110(2):270–2. 35440911 10.5195/jmla.2022.1271 PMC9014916

[44] Verbi Software. MAXQDA 2022 [computer software]. Berlin, Germany: VERBI Software; 2021.

[45] Whittemore R, Chase SK, Mandle CL. Validity in qualitative research. Qual Health Res. 2001;11:522–37. 11521609 10.1177/104973201129119299

[46] Ryan RM, Curren RR, Deci EL. What humans need: Flourishing in Aristotelian philosophy and self-determination theory. The best within us: Positive psychology perspectives on eudaimonia. Washington, DC, US: American Psychological Association; 2013. p. 57–75.

[47] Eskin M. 27The role of culture in a suicidal process. 2024 [cited 1/30/2025]. In: Suicide Across Cultures: Understanding the variation and complexity of the suicidal process across ethnicities and cultures [Internet]. Oxford University Press, [cited 1/30/2025]; [0]. Available from: 10.1093/med/9780198843405.003.0003.

[48] Atilola O, Ayinde O. A cultural look on suicide: the Yorùbá as a paradigmatic example. Mental Health, Religion & Culture. 2015;18(6):456–69.

[49] Jensen SKG, Murray SM, Placencio-Castro M, Kajani U, Amponsah D, Sezibera V, et al. Family violence reduction within a parenting intervention in Rwanda: a mixed-methods study. Pediatrics. 2023;151(Supplement 2):e2023060221L. 37125890 10.1542/peds.2023-060221L

[50] Jigsaw. What does One Good Adult mean? 2021 [Available from: https://jigsaw.ie/what-does-one-good-adult-mean/.

[51] Gureje O, Nortje G, Makanjuola V, Oladeji BD, Seedat S, Jenkins R. The role of global traditional and complementary systems of medicine in the treatment of mental health disorders. The Lancet Psychiatry. 2015;2(2):168–77. 26359753 10.1016/S2215-0366(15)00013-9 PMC4456435

[52] Long M. ‘We’re not monsters … we’re just really sad sometimes:’ hidden self-injury, stigma and help-seeking. Health Sociology Review. 2018;27(1):89–103.

[53] Gratz KL, Roemer L. Multidimensional assessment of emotion regulation and dysregulation: development, factor structure, and initial validation of the difficulties in emotion regulation scale. J Psychopathol Behav Assess. 2004;26:41–54.

[54] O’Connor RC, Kirtley OJ. The integrated motivational–volitional model of suicidal behaviour. Philos Trans R Soc Lond B Biol Sci. 2018;373(1754):20170268. 30012735 10.1098/rstb.2017.0268 PMC6053985

[55] Markus HR, Kitayama S. Culture and the self: implications for cognition, emotion, and motivation. Psychol Rev. 1991;98:224–53.

[56] Green JY. Rwandan infant caregiving: promoting a culture of peace. In: Ashdown BK, Faherty AN, editors. Parents and caregivers across cultures: positive development from infancy through adulthood. Cham: Springer International Publishing; 2020. p. 11–29.

[57] Mesquita B. Between us: How cultures create emotions. WW Norton & Company; 2022.

[58] Joiner TE, Van Orden KA, Witte TK, Selby EA, Ribeiro JD, Lewis R, et al. Main predictions of the interpersonal-psychological theory of suicidal behavior: empirical tests in two samples of young adults. J Abnorm Psychol. 2009;118(3):634–46. 19685959 10.1037/a0016500 PMC2846517

[59] Orri M, Paduanello M, Lachal J, Falissard B, Sibeoni J, Revah-Levy A. Qualitative approach to attempted suicide by adolescents and young adults: the (neglected) role of revenge. PLoS One. 2014;9(5):e96716. 24802777 10.1371/journal.pone.0096716 PMC4011950

[60] Atilola O, Ayinde O. The interface between culture and mental health in Africa: implications for clinical practice and research. Transcult Psychiatry. 2015;52(2):198–214.25480488

[61] Eskin M. The role of culture in non-suicidal self-injury and suicidal behavior. J Cross-Cult Psychol. 2024;55(1):25–42.

[62] Fricker M. Epistemic Injustice: Power and the Ethics of Knowing. Oxford University Press; 2007.

[63] Smith LT. Decolonizing Methodologies: Research and Indigenous Peoples. Zed Books; 1999.

[64] Fernando S. Mental Health Worldwide: Culture. Globalization and Development. Palgrave Macmillan; 2014.

[65] Cornwall A, Jewkes R. What is participatory research? Soc Sci Med. 1995;41(12):1667–76. 8746866 10.1016/0277-9536(95)00127-s

[66] Mazanderani F, Paparini S, Papoutsi C. Narrative and the construction of illness experience. Sociol Health Illn. 2012;34(4):578–92.

[67] Ndayisenga JP, Oudshoorn A, Mukamana D, Babenko-Mould Y, Jackson KT, Hynie M. A reflection on ethical and methodological challenges encountered during a critical ethnographic study with various Rwandan society members on unintended adolescent pregnancies. Discov Public Health. 2024;21(1):84.

[68] Leventhal H, Brissette I, Leventhal EA. The common-sense model of self-regulation of health and illness. In: Cameron LD, Leventhal H, editors. The Self-Regulation of Health and Illness Behaviour. London: Routledge; 2003. p. 42–65.

