# Supplementary material for: Culturally and structurally embedded pathways to youth self-harm in Rwanda: perspectives from young people, parents, and healthcare providers
Source: BMC Psychol. 2025 Nov 22;13:1394. doi: 10.1186/s40359-025-03676-y (PMC12751344; doi:10.1186/s40359-025-03676-y)
Supplement: Supplementary file 2 — Additional file 2. [file 40359_2025_3676_MOESM2_ESM.docx]

**Additional File 1: Illustrative topic guides**

1. **Youth with experience of suicidal behaviours / suicidal thoughts / self-harm**

- **Life until now**

1. Can you tell me about your childhood? What was it like growing up? What were some of the difficult parts? And the good parts?
2. Can you tell me about the family you grew up in? What was it like? What was your relationship with your parents and siblings like?
3. Have you attended school? If yes, can you tell me about what it was like? your experiences?
4. If no, can you tell me about how you have spent your time?
5. What was your experience like in school/other activity? Did you enjoy learning? What were your favourite subjects?
6. What were some of the challenges, hardships or difficulties did you face while in school/ your early life?

- **Current Activities/Work/ Study now**

1. What are you doing now? How do you feel about your day-to-day work/other activities?
2. Have you faced any challenges or barriers in getting an education or employment?

- **Relationships and Social Life**

1. Can you describe key relationships outside of your family? These might be good or bad.
2. Relate these back to the timeline to understand when occurred. When one completed, ask “any others?”
3. How have your relationships affected your mental health and well-being?

- **Culture and Community**

1. Are there cultural or community rituals that have influenced your values and beliefs?
2. How have your experiences and community shaped your values and beliefs?

- **Feelings and wellbeing**

1. When did you first notice upsetting feelings? Relate this to the timeline.
2. What was it like to talk about feelings in your family? And with friends? Relate this to anyone named as important on the timeline.
3. How are feelings and distress talked about in the community? How are they responded to?
4. Have you or anyone close to you been impacted by mental health difficulties?

- **Reflection and Future Plans**

1. Looking back on your life so far, what are some things you are proud of or grateful for?

- **What are your hopes and goals for the future?**

1. Is there anything else you would like to share or talk about?
2. What are some of your hopes and wishes for yourself and your community?

- **Their Suicidal Thoughts and Behaviours**

1. Can you tell me what you think of when you hear the words "suicide" or "self-harm"? How do you think about these things when they come up in your life or in the community? (i.e., identity)
2. What name would you give these suicide thoughts? Probe Some people call them “Dark Thoughts, Dark Clouds, etc”?

*If the person names the suicidal thoughts differently, please use the name in the following questions*

1. Have you ever experienced --------------------? When did ---------- first visit you? When did you first experience them? How long did it go on for (i.e., identity and timeline)
2. What do you think contributed/invited ---------------(suicidal thoughts or behaviours) in your life? What led to these? (i.e., risk factors, identity and perceived caused)
3. What were your feelings after (----------------) had visited you? And your thoughts? (i.e., function and perceived consequences)
4. Sometimes some people find (-----------------) to be helpful at sometimes. Do you think (-------------) is helpful sometimes, not helpful at all or a bit of both?
5. Sometimes people describe feeling self-harm helps them cope. Can you tell us about how that was for you?
6. How did your family and community respond when they learned about (----------) to come into your life? How did your family and community respond when they learned about suicidal thoughts or behaviours? (Prompt for focus on parents) What was that like?
7. What makes it difficult for parents to understand and support young people who self-harm?
8. What do you think parents might need to help them support young people going through (---------------)?

- **Coping and Recovery (i.e., control)**

1. Have you faced or been visited with (------------------) in the past?
2. How did you respond whenever (-----------------------) visited in the past?
3. How have you coped with difficult experiences and emotions in the past?
4. Are there any strategies or coping mechanisms that have been particularly helpful for you?
5. How did you learn that these strategies helped in responding to the (---------------)?
6. Who has been with you on the journey of responding to the (--------------------)?
7. Have you received any professional help in the past for your self-harm? If so, please explain the nature of the help
8. What support did you get? What made it easier to get that support? And what made that harder?
9. What kind of support or help do you think would be helpful for someone who is experiencing suicidal thoughts or behaviours?
10. What are your hopes and goals for the future?

Invite the person to think about what they would do in future in case the (----------------) visit again?

- Print/Create a list of things they will do onto little card
- Write a quick list and give it at the end of the session to the person to go with it

1. **Youth without experience of suicidality**

- **Introduce yourself and explain the purpose of the interview.**

1. Explain the purpose of the interview and what we will be discussing difficult topics. Remind them of the section on the information sheet re: where they can seek support.
2. Can you tell me a little about yourself and how you currently spend your time?
3. Can you tell us a little be about your friends?

- **Overview of mental health**

1. What do you understand by the term mental health?
2. What are your thoughts about self-harm and suicide? Prompt for understanding of the terms and then any general initial reactions to the topic.
3. How does it feel to talk about this topic?

- **Views of self-harm and suicide**

1. Can you describe any contact you have had with young people who have harmed themselves?
2. What do you think makes a young person hurt themselves? What does it do for them?
3. What do you think might make a young person kill themselves? What does it do for them?
4. How do you think young people feel after self-harming?
5. How do you think young people feel after trying to kill themselves?
6. Sometimes people describe that self-harm helps them cope. What do you think about this?

- **Support**

1. How would you respond to a friend who had harmed themselves? Or tried to kill themselves?
2. What kind of support or help do you think would be helpful for someone who is experiencing suicidal thoughts or behaviours?
3. What could they do to help themselves?
4. What might stop a young person from getting support about suicide and/or self-harm? What might help them?

- **Parents’ response**

1. How do you think parents respond?
2. What makes them respond like that?
3. What impact do you think that has on the young person?
4. What makes it difficult for parents to understand and support young people who self-harm?
5. What do you think parents might need to help them support young people?

- **Final questions**

1. Is there anything else you’d like to tell us about your views about self-harm and suicide in young people?
2. **Parents/carers with experience of youth with suicidal behaviour/ thoughts / self-harm**

- **Prompts**

1. Can you tell us a little bit about yourself and your family? Prompt to focus on their young person.
2. Can you tell us a little bit about what happened with your young person? Focus on mental health
3. When did you become aware that your young person was harming themselves / thinking about ending their life?

- **Ask about how many times their young person harmed self – being sensitive to possibility of suicide.**

1. What do you know about the specific situation that led them to harm themselves?
2. What were your thoughts and feelings at the time?
3. How do you think they were feeling before (and if appropriate, after) they harmed themselves?
4. What did you do when they harmed themselves? What did you say to them? Focus on their response to the young person.
5. What did you do to look after yourself at that time?
6. How do you feel about it now?
7. What are the reasons, as you understand it, that they harmed themselves?
8. (if appropriate) And why did they carry on with that?
9. What do you think they found helpful to help them with self-harm?
10. Have they received any support for this? Ask about various types of support – professional, family, church etc.
11. What was useful about that support for them?
12. What challenges were there in getting support for them?
13. And what about you – how has this affected you?
14. What kind of support would you like for yourself? Explore barriers and ideas against and for getting support.
15. Do you have any further comments to help us understand your views?
16. **Parents without experience of youth with suicidal behaviour /thoughts / self-harm from the community**

- **Prompt questions**

Opening

- What are the major challenges for young people (? define age range?) that are facing these days?
- How do you or people in your community understand “mental health”? What does this cover? What might be linked to better mental health? And worse mental health?
- What are the risks for young people’s mental health these days?
- **“Self-harm”**
- Why might a young person harm themselves?
- What response do adults in the community (e.g. family, teacher, neighbour, church member etc) generally have to finding out a young person is harming themselves? (give appropriate examples of self-harm)? Probe for their behaviour, understanding, help-seeking, and support/response given to the young person?
- How might a parent feel if their young person is harming themselves? Probe for both concern about the young person and impact on the parent themselves.
- **“Suicide”**

1. Why might a young person try to end their life?
2. What about the response from adults in the community to finding out a young person has tried to end their lives? Probe for their behaviour, understanding, help-seeking, and support/response given to the young person?
3. How might the parent feel if their young person has tried to end their life? Probe for bother concern about the young person and impact on the parent themselves

- **Resources**

1. What support does the young person need? What support does the parent need? What information does the community need?
2. What is there in the community to support people? What else is needed?
3. **Healthcare providers and community leaders**

**Opening**

1. Please describe your role in relation to youth and family mental health

**Views and understanding (including function and risks)**

1. We are going to ask you about suicide, in relation to killing oneself. We will then ask about self-harm, whereby a person intentional physically harms themselves.
2. In your view, what leads a young person to think about suicide? And to act on that? How might it provide some relief to the young person? What is the purpose of it for the young person? What effect does it have on their life? What risks are there immediately, and long-term?
3. In your view, what leads a young person to self-harm? How might it provide some relief to the young person? What is the purpose of it for the young person? What effect does it have on their life? What risks are there immediately, and long-term?
4. Are there cultural practices that might help in supporting young people experiencing suicide?

**Observations of youth and parents’ perceptions**

1. From your work, what do you notice about how young people think and feel about suicide and self-harm?
2. From your work, what do you notice about how parents and other caregivers think and feel about suicide and self-harm?
3. How do you think parents respond to their young people when they found out about the young person’s suicide or self-harm? How do those responses impact the young person?

**Needs, current and future support**

1. What do young people need to help them with suicide? And self-harm?
2. What do their parents’ / caregivers need?
3. What existing support are you aware of for young people in relation to suicide? And self-harm?
4. What existing support is there for parents and other caregivers?
5. What is missing from that support? Are there any plans you are aware of to address these issues?

**Final question**

Is there anything else you’d like to share with us about this topic?
