# Supplementary material for: Culturally and structurally embedded pathways to youth self-harm in Rwanda: perspectives from young people, parents, and healthcare providers
Source: BMC Psychol. 2025 Nov 22;13:1394. doi: 10.1186/s40359-025-03676-y (PMC12751344; doi:10.1186/s40359-025-03676-y)
Supplement: Supplementary file 3 — Additional file 3. [file 40359_2025_3676_MOESM3_ESM.docx]

**Additional File 3: Reflexive Methodology Account**

This supplementary document provides a brief account of the reflexive and methodological practices we utilised to support conduct our qualitative study in Rwanda. It arises from team discussions, consultative meetings, shared reflexive notes, and analytic debates conducted throughout the research process. Our aim is to offer transparency regarding how cross-cultural, ethically sensitive qualitative research was operationalised, and to share practical strategies and adaptations that may inform future research practice. Themes running throughout our approach were attempts to maintain reflexive practice, epistemic humility, and an iterative, collaborative approach. We do not suggest that our project was free from the influence from colonialism, issues around power, dominance of Western thinking and so on. Rather, we provide a summary of how we intentionally sought to work with and address these challenges. Our contribution is offering, via this practice-based account, a description of how we addressed this critical element of research across cultures, particularly relevant to qualitative work.

We cover six topics:

1. Conceptualization of the research
2. Team composition and reflexive approach
3. Team development
4. Analytical process and managing interpretive tensions
5. Validation work and participatory adaptation
6. Ethical reflexivity and narrative representation

We summarise our key actions and their purpose in Additional File 3: Table 1.

1. **Conceptualisation of the research**

The project conception process was rooted in a series of iterative conversations and digital interactions between authors FM, VS, and JK. They held introductory online meetings, discussed the ongoing research activities and the gaps existing. This initial engagement between the researchers from Rwanda and FM was not simply logistical but reflexive in nature. It involved critical dialogue on positionality, language, and research priority areas on the topic. This led to research questions developed not from one-sided assumptions, but from shared concerns and complementary expertise. The team co-developed research questions and methodological priorities that were both contextually grounded and informed by scientific evidence. This issue is core to decolonising research, to avoid externally-imposed research priorities [1]. An extension to this would have been to talk directly with communities, to create more fully participatory approach [2], however the tension here is how to do this in an ethical fashion prior to acquiring funding to cover expenses incurred by people in those communities.

1. **Team composition and reflexive approach**

Our wider team was made up of a range of academics and a policymaker, from students gaining apprenticeship experience, through early and mid-career academics, to an established Professor as well as a policymaker. Further, we were composed of psychology students and registered psychologists from the UK, Rwanda, France, Malaysia and Malawi. This range of cultural backgrounds was intentional, with the idea this may facilitate uncovering of assumptions held due to culture. This contributed to avoiding a single-focused, potentially colonial viewpoint of the research subject [1]. The grant funding this project was awarded to the UK lead (FM), however was developed with the Rwandan colleagues (as above). The UK lead has been responsible for leading the outputs, however with discussion, input, additions and development with the team.

To manage the important issues that may arise owing both to culture but also power (for example, seniority, who holds the funding), we worked to facilitate the habit of discussion, partly through team development (see below), but also through recognition of the value to our analysis to uncovering assumptions. We worked to commit to an open, reflexive approach throughout our work [2]. We recognised that reflexivity was not something we could “do” and move on, rather a series of opportunities to consider, change practice, and a goal to work towards.

This practice of examining our positions continued as we refined the data collection tools, working to each understand why some questions were present, their perceived purpose and value, appraisal of the need to reword or reconsider questions, and the assumptions that they rested on which may not have been explored. Collaboration here was crucial to provide relevant interview questions, that allowed us to address our research aims [3].

In addition, during discussion of the data at an early stage, we talked about which elements of the data had surprised us or anything that had seemed particularly unremarkable (for example by thinking about the areas we each felt less inclined to write about as they seemed less interesting to us). This helped us discuss our different reactions and weight given to the described experiences of abuse and hardship, for example. This led to discussion of ideas about why and how these may link to self-harm with curiosity. This practice of self-reflection and openness aided our ability to integrate viewpoints and understandings [4].

1. **Team development**

Intentional steps were taken early in the project to develop as a team. This was viewed as methodological groundwork. Cultural ice-breaking activities and informal discussions allowed team members to share elements of their cultural backgrounds, experiences, and everyday practices. After work walks, and ice-breaking games allowed us to develop a sense of safety together, through trust and sharing food and laughter as a basis for building relationships. Playing games that were somewhat “silly” helped us to see one another as people. For FM and SW, there was a reflection on how this felt strange as differed from their typical working practices of getting straight to tasks, however our reflection is that this time allowed a sense of team to develop, facilitating some of the later discussions.

From the outset, we encouraged openness about discomfort and disagreement. Aware of the potential “elephants in the room”, we sought shared agreement to discuss issues and talked about “big” topics such as how colonialism had impacted Rwanda and could impact our project. Over time we talked about various issues, including how Rwandan practices of drumming, storytelling, and communal ceremonies, are living practices rather than cultural curiosities. Conversations also addressed the risk of Western exoticisation of these practices [3], but the reaction against that could be to simply ignore these parts of life, and how subtle forms of "othering" could enter even well-intentioned research [5]. We explicitly discussed welcoming questions, challenges, requests for clarification and alternative viewpoints. Additionally, subtle things such as accent and language were also discussed and the ways these can be addressed during the conversations.

This investment in building a reflexive and critical team space helped to surface hidden assumptions early, build trust across cultural and disciplinary differences, and create a relational foundation [6] for collaborative and ethically grounded analysis. Power and hierarchy were not eliminated, however we took intentional steps to reduce their impact.

1. **Analytical process and managing interpretive tensions**

No coding frame was imposed on the data, rather we engage in open coding, to work to focus on listening to the participants’ voices and knowledge [5], however acknowledging our biases would be present. During data analysis, we worked first in pairs and then together as a group on the data. In all cases, interpretive tensions were present. For example, differences emerged over whether emotional suppression should be read as evidence of severe distress or as a culturally appropriate mode of managing emotion; and as to the role of poverty as being linked to self-harm. We noted differences regarding explanatory models of distress. Some interpretations leaned towards a more biomedical framings, while others highlighted trauma, or relational, spiritual, and social causes. Discussions around these differences helped refine the thematic structure and enabled a more multi-layered analysis that better reflected the complex and embedded nature of self-harm experiences in context.

Rather than seeking to rush to agreement, these tensions were treated as prompts for deeper enquiry. Working to remain curious, discussions explored where the viewpoints came from, and what they meant about how we might understand the data. To support this, the original Kinyarwanda transcripts were revisited collaboratively by bilingual team members. This process ensured that the analysis remained anchored in participants’ own language and conceptual framings.

By holding space for disagreement and discomfort, we were able to explore how we made sense of the data in more detail, providing an analysis based on multiple perspectives.

1. **Community validation work and participatory adaptation**

Member checking or validation sessions provided further insights into power dynamics and communication challenges, here outside our immediate team. Early validation sessions showed that the health professionals, policymakers and community leaders shared their views readily, however the parents and young people attending formal meetings with formal presentations of research findings did not. On reflection, this is something we could have predicted and we noted the extent to which we were responding to our timetabling needs in planning those sessions.

To address this, to encourage greater engagement from the parents and young people, we adapted the member checking approach to a drama-based format using composite, anonymised scenarios derived from the data. Short scenes were created reflecting common experiences, such as family conflict, community stigma, or struggles with emotional distress. This drama format allowed participants to engage with the material without personal disclosure. Facilitators then guided group discussions, inviting reflections on the situations presented, and encouraging alternative interpretations and responses. This process of using drama was more familiar to the young people, in particular. We gained insight from them about the findings, particularly regarding important areas where young people’s and parents’ views diverged, and ideas for intervention. Further, this was vital to avoid wholly doing research “to” our participants, towards involving and doing with [1].

Adapting the validation process in this way highlighted the need for flexible, participatory methods that prioritise delivering material in a manner that is most engaging, rather than feeling constrained by the need to use more formal, academic formats.

1. **Ethical reflexivity and narrative representation**

Given the highly sensitive nature of the topic, the ethical representation of participant narratives was a concern throughout the project. We agreed that quotes were important to illustrate the analysis, and consent from participants had covered this issue. However, it was clear for ethical reasons that this should be done in such a way that did not identify anyone.

When deciding whether to include powerful but deeply personal accounts, we considered whether the analytic value outweighed the potential risk of re/traumatisation, inadvertent stigmatisation, or simply unnecessary details that might be voyeuristic. We were conscious of representing our participants as victims and of inadvertently reinforcing stigmatising stereotypes [1, 3]. In some cases, powerful quotations were excluded, particularly where participants had described trauma and/or abuse. This was largely to preserve participant dignity and anonymity. We considered that the participant had used the choice they felt they had during the interviews to voice these experiences, and that was an argument for including more details. We reflected on the risk of being overly paternalistic in seeking to protect the participants. We considered the desire to not present excessively distressing material to the reader, with the observation that many likely readers were in the position where such trauma was unlikely to be part of their lives. This was a complex decision, and a subject for further debate and discussion. We sought however to balance upholding ethical standards with maintaining trustworthiness in the final presentation of the findings.

**Additional File 3: Table 1** Summary of reflexive practice actions

| **Research stage** | **Reflexive practice** | **Purpose** |
| --- | --- | --- |
| Conceptualisation of research | Explore initial research ideas together, with space for mutual sharing of ideas. | Ensuring relevance of research and sharing power. |
| Team composition and reflexive approach | Creating a diverse team, covering multiple areas of expertise and viewpoints. Establishing commitment to reflexive approach and open communication. | Ensure range of viewpoints to help broaden analysis and relevance. Foreground importance of reflexivity. |
| Team development | Building trust, clarifying positionality, sharing food and laughter. | Establish relational safety, addressing power imbalances. |
| Data collection tool development | Examining the initial tools to ensure aims can be met. Critique tools, reword and test. | Avoid cultural imposition wording and conceptual focus, work with local expertise. |
| Data analysis | Embracing disagreement, open discussion, revisiting transcripts. | Enable shared meaning-making and interrogation of assumptions. |
| Validation / member checking of data | Tailor to the context of participants and preferences/familiar modes of communication, e.g. participatory drama, storytelling. | Facilitates inclusive feedback from participants to enhance member checking. |
| Ethical reflexivity and narrative representation | Careful consideration of when and how to include participants’ quotes, debate and discussion of pros and cons. | Maintain dignity of participants and ethical storytelling . |

**References**

1. Denscombe M. Decolonial research methodology: an assessment of the challenge to established practice. International Journal of Social Research Methodology. 2025;28(2):231-40.

2. Keikelame MJ, and Swartz L. Decolonising research methodologies: lessons from a qualitative research project, Cape Town, South Africa. Global Health Action. 2019;12(1):1561175.

3. Broesch T, Crittenden AN, Beheim BA, Blackwell AD, Bunce JA, Colleran H, et al. Navigating cross-cultural research: methodological and ethical considerations. Proc Biol Sci. 2020;287(1935):20201245.

4. Ross K, Li P. Beyond Reflexivity: Centering Recognition and Relational Dialogue in Social Inquiry. Cultural Studies ↔ Critical Methodologies. 2025;25(2):133-43.

5. Thambinathan V, Kinsella EA. Decolonizing Methodologies in Qualitative Research: Creating Spaces for Transformative Praxis. International Journal of Qualitative Methods. 2021;20:16094069211014766.

6. Gerlach A. Thinking and Researching Relationally:Enacting Decolonizing Methodologies With an Indigenous Early Childhood Program in Canada. International Journal of Qualitative Methods. 2018;17(1):1609406918776075.
